# Can You Pass the Acid Test? Critical Review and Novel Therapeutic Perspectives of Δ^9^-Tetrahydrocannabinolic Acid A

**DOI:** 10.1089/can.2016.0008

**Published:** 2016-06-01

**Authors:** Guillermo Moreno-Sanz

**Affiliations:** Department of Anatomy & Neurobiology, School of Medicine, University of California Irvine, Irvine, California.

**Keywords:** THCA-A, THC, THC-COOH

## Abstract

Δ^9^-tetrahydrocannabinolic acid A (THCA-A) is the acidic precursor of Δ^9^-tetrahydrocannabinol (THC), the main psychoactive compound found in *Cannabis sativa*. THCA-A is biosynthesized and accumulated in glandular trichomes present on flowers and leaves, where it serves protective functions and can represent up to 90% of the total THC contained in the plant. THCA-A slowly decarboxylates to form THC during storage and fermentation and can further degrade to cannabinol. Decarboxylation also occurs rapidly during baking of edibles, smoking, or vaporizing, the most common ways in which the general population consumes Cannabis. Contrary to THC, THCA-A does not elicit psychoactive effects in humans and, perhaps for this reason, its pharmacological value is often neglected. In fact, many studies use the term “THCA” to refer indistinctly to several acid derivatives of THC. Despite this perception, many *in vitro* studies seem to indicate that THCA-A interacts with a number of molecular targets and displays a robust pharmacological profile that includes potential anti-inflammatory, immunomodulatory, neuroprotective, and antineoplastic properties. Moreover, the few *in vivo* studies performed with THCA-A indicate that this compound exerts pharmacological actions in rodents, likely by engaging type-1 cannabinoid (CB1) receptors. Although these findings may seem counterintuitive due to the lack of cannabinoid-related psychoactivity, a careful perusal of the available literature yields a plausible explanation to this conundrum and points toward novel therapeutic perspectives for raw, unheated Cannabis preparations in humans.

## Introduction

**FIG. 1.**
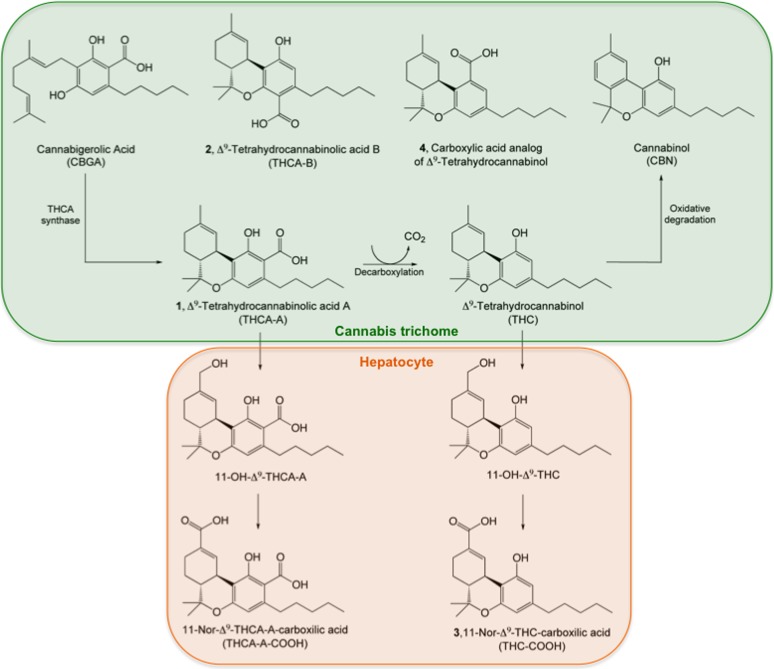
Synthetic and metabolic routes for THCA-A. THCA-A (**1**) is produced by THCA synthase from its precursor, CBGA, and stored in *Cannabis* glandular trichomes. THCA-A decarboxylates to form THC, which can further degrade to cannabinol. Other acid derivatives found in the plant are THCA-B (**2**), the minor isomer of THCA-A, and the carboxylic derivative of THC (**4**). Conversion of THCA-A into THC does not occur *in vivo*. Rather, both compounds undergo similar metabolic pathways, transforming first into the 11-hydroxyl intermediate and further oxidizing to the 11-carboxylic metabolites (THCA-A-COOH and **3**). CBGA, cannabigerolic acid; THC, tetrahydrocannabinol; THCA-A, tetrahydrocannabinolic acid A.

Δ^9^-tetrahydrocannabinolic acid A (THCA-A, 2-carboxy-THC) is the acidic precursor of Δ^9^-tetrahydrocannabinol (THC), the main psychoactive compound in *Cannabis sativa*. THCA-A is biosynthesized from cannabigerolic acid (Fig. 1) and accumulates in the glandular trichomes of flowers and leaves where it represents up to 90% of the total THC.^1,2^ As the plant reaches maturity, THCA acts as a necrosis-inducing factor, causing senescence in leaf tissues through a calcium-independent mechanism that involves the opening of mitochondrial permeability transition (MPT) pores.^3^

THCA-A slowly decarboxylates to form THC during storage and fermentation, and can further degrade to form cannabinol by effect of temperature, auto-oxidation, and light (Fig. 1). Decarboxylation also occurs rapidly during baking or smoking, the most common ways in which Cannabis is generally consumed.^4^ However, this decarboxylation is only partial and, therefore, THCA-A can be found, together with THC, in the oral fluid, serum, and urine of Cannabis consumers.^5–7^ Since THCA-A does not seem to convert to THC *in vivo* and displays its own metabolic and elimination pathways, it was proposed as a marker capable of distinguishing between the use of Cannabis and prescription synthetic THC (Marinol^®^).^8,9^

Contrary to THC, THCA-A does not elicit psychoactive effects in humans and, perhaps for this reason, its pharmacological value is often neglected. In fact, many studies vaguely use the term “THCA” to indistinctly refer to: **1**, the biosynthetic precursor, 2-carboxy-THC; **2**, its isomer, 4-carboxy-THC; **3**, the inactive metabolite 11-nor-9-carboxy-THC, which is generated in the liver by the enzymatic deactivation of THC and is commonly used as a biomarker for Cannabis consumption; and **4**, the also inactive carboxylic acid analogue of THC (Fig. 1). Despite this perception, many *in vitro* studies suggest that THCA displays a rather active pharmacological profile, which includes potential anti-inflammatory,^10^ immunomodulatory,^11^ neuroprotective,^12^ and antineoplastic^13,14^ properties. Moreover, the scarce *in vivo* studies performed with THCA-A indicate that this compound exerts pharmacological actions in rodents, likely by engaging type-1 cannabinoid (CB1) receptors.^15^

## THCA-A in the Cannabis Plant

THCA synthase is a flavoprotein that biosynthesizes THCA-A by catalyzing the stereospecific oxidative cyclization of the geranyl group within cannabigerolic acid (CBGA, Fig. 1).^16^ Since THCA-A is the precursor of THC, polymorphisms of this enzyme are responsible for the difference between “drug type” and “fiber type” Cannabis plants.^17,18^ THCA synthase localizes to the storage cavity of glandular trichomes—capitate-stalked, capitate sessile, and bulbous—within the bracts, floral leaves, leaves, and stems of the Cannabis plant,^19^ which is, therefore, not only the site for THCA-A accumulation but also for its biosynthesis. A rationale for this observation is that cannabinoid acids are cytotoxic substances, since both CBGA and THCA-A induce cell death in both Cannabis and insect cells (Spodoptera frugiperda, Sf9), suggesting that these molecules might serve to defend the plant against predators.^2^ This cell-death mechanism occurs through a necrotic pathway involving mitochondrial dysfunction and opening of MPT pores, although its relevance to mammalian cells remains unclear.^3^

THCA synthase also represents an attractive target for the biotechnological production of THC. Because CBGA is easy to synthesize,^20^ and THCA-A readily decarboxylates into THC by heating, substantial efforts were aimed at developing a suitable expression system for THCA synthase. Two different cost- and fermentation-friendly expression systems were initially established: (i) transgenic tobacco hairy roots^20^ and (ii) methylotrophic yeast *Pichia pastoris*.^21^ Although early studies showed only insignificant amounts of active biocatalyst,^21^ two independent groups recently reported the successful, milligram-scale synthesis of THCA-A in *Pichia pastoris*.^22,23^

## Can You Pass the Acid Test?

The term “tetrahydrocannabinolic acid” or “THCA” has been hazily used in the literature to refer to several acidic derivatives of THC, making it confusing to clearly individuate their physiological and pharmacological profile. In 1965, Professor Friedhelm Korte from the University of Bonn was the first to identify *tetrahydrocannabinolcarboxylic acid* (2-carboxy-THC, **1**, Fig. 1) as a major component of hashish.^24^ Four years later, in 1969, Raphael Mechoulam et al. from the University of Jerusalem reported the existence of a second THC acid, the isomer 4-carboxy-THC (**2**, Fig. 1), and named the former THCA-A and the latter THCA-B.^25^ THCA-B was only found in hashish samples that contained very little or no THCA-A, and its overall concentrations were generally lower than 0.5% in weight. Subsequent studies, however, were not able to confirm the occurrence of THCA-B.^26^ A year later, in 1970, the existence of acid metabolites of THC was reported by Agurell et al., who isolated 11-nor-9-carboxy-delta 9-THC (THC-COOH, **3**, Fig. 1) from an acidic fraction in the urine of rabbits treated with radiolabeled THC.^27^ Further studies confirmed the importance of this metabolic route, demonstrating that THC-COOH produces no psychotropic responses in humans, and is only further metabolized into glucuronide conjugates.^28^ Accordingly, THC-COOH does not elicit cannabimimetic behaviors in mice and shows no affinity toward the CB1 receptor.^29^ Finally, investigators from the University of Mississippi recently reported the presence of a carboxylic derivative of THC (**4**, Fig. 1) in high-potency *Cannabis sativa* plants.^30^ This compound, improperly referred to as *Δ9-tetrahydrocannabinolic acid*, displayed low affinity (in the milli-molar range) for both CB1 and CB2 receptors. This is in agreement with a previous report, where compound **1** was synthesized as part of a structure–activity relationship study conducted on the C-1 position of THC.^31^

## Does THCA-A Bind to Cannabinoid Receptors?

Three studies have attempted to answer this question by testing *in vitro* the affinity of THCA-A toward CB1 and CB2, yielding contradictory results. In 2006, Verhoeckx et al. reported THCA-A to be a weak agonist of CB1 and CB2 receptors compared with THC (Ki_CB1_=630 vs. 3.5 nM; Ki_CB2_=890 vs. 3.2 nM).^11^ The authors mentioned that affinity values were determined by binding assays using membranes isolated from Sf9 cells stably expressing human CB1 receptors, but neither the actual data nor the experimental procedure was included in the article. In 2008, Ahmed et al. isolated 11 novel cannabinoid esters from high-potency *Cannabis sativa*, 8 of which were derivatives of THCA-A. The authors clearly stated: “Both the esters and the parent acids were not active, as indicated by a CB1 receptor binding assay.”^32^ Again, neither the experimental conditions of the assay nor the affinity values for any compound were reported in the text or referenced. More recently in 2014, Rosenthaler et al. tested seven different phytocannabinoids, including THCA-A, for their ability to bind CB1 and CB2 receptors *in vitro*. The binding experiment measured the competitive displacement of the radioligand [3H] CP-55,940 (1 nM) by several concentrations of the phytocannabinoids in membranes prepared from Sf9 cells expressing either CB1 or CB2. The study concluded that THCA-A effectively binds to both cannabinoid receptors, displaying a higher affinity for CB1, with Ki values of 23.51±3.5 nM and 56.13±8.2 nM, respectively. In fact, THCA-A (logIC_50_=1.793±0.00) and THC (logIC_50_=1.941±0.01) displaced CP-55,940 from CB1 in a similar range of concentrations.^33^

This finding is in agreement with the results obtained in the single *in vivo* preclinical study performed with THCA-A to date.^15^ In 2013, Rock et al. found that THCA-A attenuated nausea-induced gaping in rats and vomiting in shrews through a mechanism that required CB1 activation. The effect could be reverted by the coadministration of SR141716, a selective CB1 antagonist. The authors acknowledged in their discussion the discrepancy between their observation and the *in vitro* data available at the time, since the study by Rosenthaler et al. was not yet published. They even ruled out the possibility of THCA-A converting to THC and activating CB1, a transformation previously suggested not to occur *in vivo*,^8^ by confirming (i) the absence of THC in blood and (ii) the lack of cannabimimetic responses, like hypothermia or reduced locomotion, after THCA-A injection in rats.^15^

Based on the results suggesting that THCA-A binds to CB1 receptors *in vitro* with an affinity similar to that of THC, a plausible explanation to these contradictory observations could be that THCA-A is a CB1 agonist with restricted access to the central nervous system (CNS). If this were true, THCA-A would still be able to activate peripheral, but not central, CB1 receptors. Three different lines of evidence support this hypothesis: (i) peripheral mechanisms were shown to contribute to CB1-mediated antiemetic actions^34–37^; (ii) THC causes reductions in body temperature and motor activity by engaging CB1 receptors in central brain areas, such as the hypothalamus and basal ganglia^38,39^; and (iii) brain disposition of THC is augmented in knockout mice lacking P-glycoprotein (P-gp/abcb1) and/or breast cancer resistance protein (Bcrp/abcg2).^40^ Both abcb1 and abcg2 belong to the ATP-binding cassette (ABC) family of efflux transporters and are critical to blood–brain barrier (BBB) function, where they impede the passage of their substrates to the brain.^41^ Addition of polar residues, such as a carboxylic group, to the scaffold of abcb1/abcg2 substrates markedly decreases CNS penetration,^42^ which could be the case for THCA-A. Interestingly, brain distribution has been reported for several phytocannabinoids but not for THCA-A.^43,44^ Therefore, further studies are required to confirm the ability of THCA-A to activate peripheral CB1 receptors *in vivo* and to characterize its central distribution.

## Pharmacological Actions of THCA-A

Several groups have investigated the therapeutic potential of minor cannabinoids present in *Cannabis sativa*. These compounds may be clinically useful due to the lack of unwanted psychotropic effects.^45^ Table 1 summarizes the molecular targets reported to interact with THCA-A. Cell-based experiments suggest that THCA-A might exert (i) immunomodulatory, (ii) anti-inflammatory, (iii) neuroprotective, and (iv) antineoplastic effects. For example, THCA-A inhibits the release of tumor necrosis factor alpha (TNF-α) from LPS-activated U937 macrophages and peripheral blood macrophages in a dose-dependent manner. Verhoeckx et al. also demonstrated that THCA-A, but not THC, inhibits the enzymatic activity of phosphatidylcholine-specific phospholipase C (PC-PLC), suggesting that both compounds may exert their immunomodulatory effects through different pathways.^11^ Several phytocannabinoids, including THCA-A, weakly inhibit cyclooxygenase enzymes (COX-1 and COX-2) in a high concentration range (mM), compared with nonsteroidal anti-inflammatory drugs (NSAIDs).^10^ In the same study, THCA-A did not display COX selectivity (COX-1/COX-2 IC_50_ ratio was 2.69, Table 1). Not surprisingly, THCA-A failed to significantly inhibit prostaglandin production in TNF-α-stimulated HT29 cells at the dose tested (10% inhibition, 62.5 μM), which was 10 times smaller than the reported IC_50_. In an established *in vitro* model of Parkinson disease, treatment with THCA-A was neuroprotective against 1-methyl-4-phenyl pyridinium (MPP^+^) toxicity, increasing cell survival and markedly ameliorating altered neurite morphology at the highest dose tested (10 μM).^12^ Although the underlying mechanism is unknown, this effect is unlikely to be mediated by the free-radical scavenging potential of THCA-A. Finally, THCA-A reduces cell viability in different prostate carcinoma cell (PCC) lines, both androgen-receptor positive (LNCaP, 22RV1) and androgen-receptor negative (DU-145, PC-3). When incubated in the absence of serum, THCA-A exhibited good efficacy and potency at reducing PCC viability in the 3-(4,5-dimethylthiazol-2-yl)-2,5- diphenyltetrazolium bromide (MTT) assay.^14^ This agrees with previous results by the same group, where THCA-A was also efficacious at inhibiting proliferation of two different human breast carcinoma (HBC) cells, triple-negative MDA-MB-231 and HER2-negative MCF-7.^13^ Although activation of CB1 receptors inhibits tumor growth and metastasis of breast cancer,^46^ the mechanism underlying the antineoplastic actions of THCA-A remains unknown. Interestingly, PC-PLC is involved in maintaining the mesenchymal-like phenotype in HBC cells, where its expression is abnormally elevated.^47^ Further studies are needed to assess the potential of THCA-A to promote HBC cell differentiation by deactivating PC-PLC deactivation, possibly enhancing the effectiveness of antitumor treatments.

**Table 1.** **Molecular Targets for THCA-A**

Route	Target	Action	*EC_50_ (μM)*	Assay	Reference
Phospholipids metabolism	PC-PLC	Inhibitor	50 (aprox)	LPS-activated U937 macrophages homogenate analyzed with AmplexR Red PC-PLC assay Kit	Verhoeckx et al.^11^
Prostaglandins metabolism	COX-1	Inhibitor	1700	Conversion of [^14^C]-arachidonic acid into ^14^C-labeled prostaglandins by purified COX-1	Ruhaak et al. ^10^
	COX-2	Inhibitor	630	Conversion of [^14^C]-arachidonic acid into ^14^C-labeled prostaglandins by purified COX-2	
Transient receptor potential (TRP) channel signaling	TRPA1	Agonist	2.7±0.9	Increase of [Ca^2+^]_i_ in rTRPA1-HEK-293	De Petrocellis et al. ^14^
	TRPM8	Antagonist	0.15±0.02	Blockade of icilin (0.25 μM)-induced increase of [Ca^2+^]_i_ in rTRPM8-HEK-293	
	TRPV1	Antagonist	19.2±5.3	Blockade of capsaicin (0.1 μM)-induced increase of [Ca^2+^]_i_ in hTRPV1-HEK-293	
	TRPV2	Agonist	18.4±0.9	Increase of [Ca^2+^]_i_ in rTRPV2-HEK-293	
AEA metabolism	AEA transport	Inhibitor	>25	Blockade of [^14^C]-AEA uptake on rat basophilic leukemia cells	De Petrocellis et al. ^14^
	FAAH	Inhibitor	>50	Blockade of the enzymatic hydrolysis of [^14^C]-AEA using membranes prepared from rat brain	
	NAAA	Inhibitor	>50	Blockade of the enzymatic hydrolysis of [^14^C]-palmitoylethanolamine in hNAAA-HEK-293	
2-AG metabolism	DGLα	Inhibitor	27.3±1.6	Blockade of the hydrolysis of 1-[^14^C]-oleoyl-2-AG in COS-7 cells over expressing hDGLα	De Petrocellis et al. ^14^
	MGL	Inhibitor	46.0±1.2	Blockade of the hydrolysis of [^3^H]-2-AG using the cytosolic fraction of wild-type COS cells	

Although the ability of THCA-A to bind to cannabinoid CB1 and CB2 receptors *in vitro* remains controversial, other molecules involved in lipid metabolism have been identified as potential targets. As reported by De Petrocellis et al., 2011, THCA-A is the most potent TRPM8 antagonist of all phytocannabinoids tested. Also, it may influence endocannabinoid metabolism by inhibiting DGLα and MGL, the enzymes responsible for the synthesis and deactivation of 2-AG. On the contrary, THCA-A is not active toward the enzymes involved in the metabolism of the endocannabinoid anandamide (AEA) and other structurally related fatty acid ethanolamides.

THCA-A, tetrahydrocannabinolic acid A.

## THCA-A in Humans

Although THCA-A is described as pharmacologically inactive,^4^ reports of popular medicinal use of unheated Cannabis preparations show pharmacological effects often accompanied with a low rate of adverse psychotropic effects. For example, a large number of patients claim medical benefits from consuming Cannabis tea, in which THCA-A is usually the most abundant cannabinoid present.^48^ Even when smoked, THCA-A is only partially transformed to THC. The conversion rates during smoking range from a maximum of 70% under optimized analytical conditions (temperatures higher than 140°C) to simulated smoking processes, where only 30% of the spiked THCA-A was recovered as THC.^7^ Although its absorption from *Cannabis* smoke is expected to be minimal, THCA-A can be detected in serum, urine, and oral fluid of Cannabis consumers up to 8 h after smoking.^5,6^ For this reason, THCA-A was also investigated as a potential biomarker of Cannabis use hoping that it could potentially allow for a more accurate estimation of the time of Cannabis consumption than THC-COOH or THC.^5^ Also, detection of THCA-A could undoubtedly differentiate between the intake of Cannabis products and prescribed THC medication (i.e., Marinol), which contains only pure THC. However, THCA-A was found to have a partition coefficient similar to THC and THC-COOH, and its blood levels not to correlate with the degree of impairment stated in police and medical reports.^9^ These findings are clinically relevant, especially considering that unheated *Cannabis sativa* extracts present a markedly different pharmacokinetic and metabolic profile in healthy human subjects compared with either decarboxylated products or pure THC.^49^

## Conclusions

THCA-A is the nonpsychoactive precursor of THC, the major active component of *Cannabis sativa*, the most commonly used illegal substance in the world. Despite its lack of psychoactivity, THCA-A is not pharmacologically inactive. Many *in vitro* studies have demonstrated its capacity to interact with several molecular targets, suggesting that it might exert anti-inflammatory, immunomodulatory, neuroprotective, and antineoplastic actions. In humans, THCA-A is orally available and might contribute to modify the absorption and/or the metabolism of THC on unheated Cannabis preparations. However, its ability to bind to cannabinoid receptors remains controversial, even though *in vivo* studies seem to indicate that THCA-A is able to functionally activate CB1. Further experiments will be required to clarify this particular aspect. The ability of THCA-A to access the brain after systemic administration also remains unknown. Based on the present evidence, THCA-A access to the CNS may be restricted due to its interaction with the BBB. Such findings could have major implications for the medical use of raw, unheated Cannabis preparations, potentially allowing patients to maximize therapeutic gain on the treatment of several ailments, such as chemotherapy-induced nausea, pain, inflammation, or muscle spasticity, while limiting the undesirable psychoactive side effects of marijuana.

## Abbreviations Used

ABC: ATP-binding cassette
BBB: blood-brain barrier
CB1: type-1 cannabinoid
CBGA: cannabigerolic acid
CNS: central nervous system
HBC: human breast carcinoma
MPP+: 1-methyl-4-phenyl pyridinium
MPT: mitochondrial permeability transition
NSAIDs: nonsteroidal anti-inflammatory drugs
PC-PLC: phosphatidylcholine-specific phospholipase C
THC: Δ^9^tetrahydrocannabinol
THCA-A: Δ^9^-tetrahydrocannabinolic acid A
THC-COOH: 11-nor-9-carboxy-delta 9-THC
TNF-α: tumor necrosis factor alpha
